# Surgery After Conversion Therapy With PD-1 Inhibitors Plus Tyrosine Kinase Inhibitors Are Effective and Safe for Advanced Hepatocellular Carcinoma: A Pilot Study of Ten Patients

**DOI:** 10.3389/fonc.2021.747950

**Published:** 2021-10-19

**Authors:** Wenwen Zhang, Bingyang Hu, Jun Han, Zhanbo Wang, Guangyu Ma, Huiyi Ye, Jing Yuan, Junning Cao, Ze Zhang, Jihang Shi, Mingyi Chen, Xun Wang, Yinzhe Xu, Yanshuang Cheng, Lantian Tian, Hongguang Wang, Shichun Lu

**Affiliations:** ^1^ Faculty of Hepato-Pancreato-Biliary Surgery, Chinese People’s Liberation Army (PLA) General Hospital, Beijing, China; ^2^ Institute of Hepatobiliary Surgery of Chinese PLA, Beijing, China; ^3^ Key Laboratory of Digital Hepatobiliary Surgery, PLA, Beijing, China; ^4^ Department of Pathology, the First Medical Center of PLA General Hospital, Beijing, China; ^5^ Department of Nuclear Medicine, the First Medical Center of PLA General Hospital, Beijing, China; ^6^ Department of Radiology, the First Medical Center of PLA General Hospital, Beijing, China; ^7^ Organ Transplant Center, the Affiliated Hospital of Qingdao University, Qingdao, China; ^8^ Medical School of Chinese PLA, Beijing, China; ^9^ National Cancer Center/National Clinical Research Center for Cancer/Cancer Hospital, Chinese Academy of Medical Sciences and Peking Union Medical College, Beijing, China; ^10^ Department of Hepatopancreatobiliary Surgery, Affiliated Hospital of Qingdao University, Qingdao, China

**Keywords:** hepatocellular carcinoma (HCC), conversion therapy, surgery, systematic treatment, PD-1 inhibitors, tyrosine kinase inhibitors (TKIs)

## Abstract

**Background and Aims:**

Immunotherapy with PD-1 inhibitors combined with tyrosine kinase inhibitors (TKIs) has been proven to be effective against advanced hepatocellular carcinoma (HCC). The aim of this study was to identify the feasibility and safety of subsequent salvage surgery after this combination therapy.

**Methods and Patients:**

A retrospective analysis was performed on patients with primary HCC with major vascular invasion between 2018 and 2019. All cases were treated with a combination of a PD-1 inhibitor and TKI agents and subsequent surgery.

**Results:**

A total of 10 HCC cases with major vascular invasion met the successful conversion criteria after the combination therapy, and eight patients underwent subsequent salvage surgery after both radiology and 3D quantitative oncological assessment. Partial response (PR) was recorded in 7 of 10 patients and complete response (CR) in 3 of 10 patients before salvage surgery. Salvage surgery included right hepatectomy, left hepatectomy, and anatomic segmental hepatectomy. The mean intraoperative blood loss was 1,650 ml (50–3,000 ml). No complications beyond Clavien–Dindo level III or postoperative mortality were observed. The viable tumor cell rate of the PR cases (modified response evaluation criteria in solid tumors, mRECIST) varied from 1.5% to 100%, and only one patient had pathology-proven pathological complete response (pCR). The postoperative median follow-up time was 19.7 months (9.1–24.9 months). The 12-month recurrence-free survival rate of all cases who underwent salvage surgery was 75%.

**Conclusion:**

Salvage surgery was effective and safe after conversion therapy with PD-1 inhibitors plus TKIs and may increase the long-term oncological benefit for patients with unresectable HCC.

## Introduction

Hepatocellular carcinoma (HCC) is a global public health challenge and socioeconomic burden, with nearly 906,000 new cases every year worldwide, including over 410,000 cases in China (1, 2). The overall 5-year survival rate was only 12.5% for the HCC population in China from 2003 to 2015 (3). Although surgical treatment is the most effective curative therapy for HCC in the early stages, with a 5-year survival rate exceeding 70%, no less than 44%–62.2% of all HCC patients were initially diagnosed with advanced HCC at Barcelona Clinic Liver Cancer (BCLC) classification stage C, most of which were accompanied by portal vein tumor thrombus (PVTT) (4–7). The majority of international guidelines recommend systemic treatment rather than curative radical surgery for these patients. However, the median overall survival (OS) values of the first- and the second-line targeted therapy were only 12 and 8 months, respectively (2, 8, 9).

In recent years, immune checkpoint inhibitors (ICIs) have achieved remarkable applications in cancer treatment. However, according to previous randomized clinical trials, ICI treatment alone did not show a significant survival benefit over targeted therapy with tyrosine kinase inhibitors (TKIs) for advanced HCC (10–12). Nevertheless, recent clinical studies have reported that combining PD-1/PD-L1 inhibitors and anti-angiogenesis targeted drugs achieved an objective response rate (ORR) of 33.2%–46.0% and a disease control rate (DCR) of 72.3%–88% for the treatment of unresectable HCC (13–15). The premium outcome of median OS was over 17 months, and 8.6%–11% of the included patients achieved complete response (CR) with good treatment safety.

On the other hand, a surprising discovery was that some of the cases with CR and partial response (PR) retrieved the opportunity for surgery. Moreover, ICIs enhanced the tumor-specific immunology, which can be maintained after subsequent surgery (16). This raised the possibility that combination therapy with PD-1 inhibitors plus TKIs can be utilized as a conversion therapy for advanced HCC. However, the effectiveness, safety, and long-term oncological benefit of salvage surgery need to be clarified. Furthermore, suitable indications and operative opportunities are also required for further investigation. As the initial exploration of a prospective clinical registration study (ChiCTR1900023914), in the past 2 years, we have initiated the clinical exploration of the PD-1 inhibitor in combination with TKIs as a conversion therapy for unresectable HCC with major vascular invasion. To date, 10 patients who underwent successful conversion therapy have been reported.

## Method

### Patients

A retrospective analysis was performed on patients with primary HCC at BCLC classification stage C without extrahepatic metastasis from August 2018 to December 2019. All of these cases were treated with a first- or a second-line combination of a PD-1 inhibitor and TKI agents (in the latter case, the first-line treatment did not include a systemic treatment regimen), and salvage surgery was performed after the combination therapy with sufficient evaluation and permission of patients. The study was censored on June 30, 2021.

All patients met the following criteria prior to enrollment in the combined drug therapy protocol: 1) confirmed histologically or in accordance with the clinical diagnosis criteria of the American Association for the Study of Liver Diseases (AASLD) as HCC; 2) Child–Pugh score <7; 3) BCLC stage C, without extrahepatic metastasis; 4) Eastern Cooperative Oncology Group (ECOG) performance status (PS) score ≤1; 5) expected survival time ≥12 weeks; 6) no esophageal or gastric varicose bleeding events caused by portal hypertension occurring in the proximate 6 months; and 7) no previous anti-PD-1, anti-PD-L1/L2 antibody, anti-CTLA4 antibody, or other immunotherapy or targeted therapy with TKI agents.

The treatment regimen was recorded. In this study, the majority of patients were treated with pemlizumab and lenvatinib combination regimens. Other PD-1 inhibitor options include sintilimab and toripalimab. Other molecularly targeted drugs include apatinib (**Table 1**).

**Table 1:** Clinical characteristics of patients.

	Patient/case no.									
	1	2	3	4	5	6	7	8	9	10
**Age (years)**	56	33	48	54	43	56	62	67	61	38
**Sex**	Female	Male	Male	Male	Male	Male	Male	Female	Male	Male
**BMI (kg/m^2^)**	21.2	27.2	21.7	27.3	26.3	25.5	19.7	21.5	25.0	27.7
**ECOG PS score**	0	0	0	0	0	0	0	0	0	0
**Child–Pugh grade**	A	A	A	A	A	A	A	A	A	A
**Cirrhosis/without cirrhosis**	Cirrhosis	Cirrhosis	Cirrhosis	Cirrhosis	Cirrhosis	Cirrhosis	Cirrhosis	Cirrhosis	Cirrhosis	Cirrhosis
**Largest tumor diameter (mm)**	169.1	125.8	122.9	103.4	139.7	72.4	88.5	36.2	163.4	85.4
**Tumor number**	1	1	3	1	1	2	4	1	1	1
**BCLC stage**	C	C	C	C	C	C	C	C	C	C
**Macrovascular invasion****^a^**	VP4	VP4	VP2/IVC	VP4	VP4	VP3	VP3	VP4	VP4	VP4/LHV
**Etiology**	HBV	HBV	HBV	HBV	HBV/NAFLD	HBV	HBV	HCV	HBV	HBV
**Prior therapy**	–	–	–	–	–	TACE	–	–	–	–
**AFP (ng/ml)**	1,552.3	1,534	6,951	14.6	>60500	10.8	846.4	3.4	>60500	216.7
**Liver biopsy**	–	HCC	–	–	–	HCC	HCC	HCC	HCC	HCC
**PD-1 category**	PEM	PEM	PEM	TRI	PEM	PEM	PEM	PEM	SIN	TRI
**TKI category**	LEN	LEN	LEN	APA	LEN	LEN	LEN	LEN	LEN	LEN
**Treatment cycles** ^b^	10	4	6	6	4	6	6	5	5	6
**mRECIST**	PR	PR	PR	PR	PR	CR	PR	PR	CR	CR
**RECIST 1.1**	PR	PR	PR	PR	PR	PR	PR	SD	PR	PR

AFP, alpha fetoprotein; APA, apatinib; BCLC, Barcelona clinic liver cancer; BMI, body mass index; CR, complete response; ECOG PS, Eastern Cooperative Oncology Group performance status; HBV, hepatitis B virus; HCC, hepatocellular carcinoma; HCV, hepatitis C virus; IVC, inferior vena cava; LEN, lenvatinib; LHV, left hepatic vein; mRECIST, modified respond evaluation criteria solid tumors; NAFLD, non-alcoholic fatty liver disease; PEM, pembrolizumab; PR, partial response; RECIST 1.1, response evaluation criteria in solid tumors version 1.1; SIN, sintilimab; TACE, transcatheter arterial chemoembolization; TKI, tyrosine kinase inhibitor; TRI, toripalimab.

a Macrovascular invasion was measured in the portal vein, inferior vena cava, and hepatic vein. Portal vein invasion grade was evaluated according to the Japanese VP classification.

b Treatment cycles were the period between the first treatment cycle of PD-1 inhibitor to the last cycle before the surgery or to the last evaluation.

### Radiologic Assessment

Imaging examinations were performed before and after the combination treatment protocol. The imaging data, including enhanced magnetic resonance imaging (MRI), computed tomography (CT), and positron emission tomography computed tomography (PET-CT), were evaluated by two independent physicians using the modified response evaluation criteria in solid tumors (mRECIST) and the response evaluation criteria in solid tumors, version 1.1 (RECIST 1.1) (17). The 3D assessment of quantitative oncology utilized the total tumor volume (TTV), portal vein tumor thrombus (PVTT) volume, and future liver remnant (FLR) volume before and after the combination treatment protocol, which were quantitatively analyzed using a 3D reconstruction software (IQQA-Liver; EDDA Technology, Princeton, NJ, USA). This software was used interactively based on original CT/MRI DICOM images. For comparison purposes, FLR was set in the software as the volume of the remaining liver after hemihepatectomy where the tumor was located.

### Surgery Criteria

The criteria for successful conversion include the following: 1) Child–Pugh score <7; 2) ECOG PS score ≤1; 3) no extrahepatic lesion assessed by PET-CT (or pulmonary CT, bone scan, etc.); 4) intact vascular structure (such as the inflow and outflow) of the reserved liver: and 5) the expected ratios of FLR to standard liver volume (FLR/SLV) after resection of the tumor-bearing liver are ≥40% in compromised livers and 35% in normal livers.

All patients who met the criteria of successful conversion were informed of the benefits and risks of surgery. Patients who agreed to subsequent surgery signed written informed consent forms.

### Clinicopathological Variables

Patient-related factors included age, sex, performance status, the etiology of liver disease, presence of cirrhosis, Child–Pugh grading, and alpha-fetoprotein (AFP) level. Tumor factors included tumor number, tumor size, macroscopic vascular invasion, and BCLC staging. Surgery-related factors included intraoperative blood loss, intraoperative blood transfusion, postoperative complications, fasting time, and abdominal drainage duration. Pathological specimens were analyzed according to the seven-point baseline sampling protocol as much as possible. Patients were followed up using the serum AFP level as well as the MRI or CT of the chest and abdomen once every 2 months for 6 months and then once every 3 months afterward.

## Results

In this study, 10 patients with HCC with major vascular invasion who were eligible for surgery after PD-1 inhibitor combined with TKI agents were examined between August 2018 and December 2019. Among these patients, eight underwent salvage surgery. Another two patients met the criteria of successful conversion, but refused surgical treatment and were willing to continue with the original treatment protocol.

### Clinical Characteristics and Conversion Therapy

The pretreatment baseline level and treatment plan of the patients were recorded, as shown in **Table 1**. All patients had hepatitis-related cirrhosis [nine hepatitis B virus (HBV) and one hepatitis C virus (HCV)], and one had non-alcoholic fatty liver disease (NAFLD). All cases were evaluated at a Child–Pugh grade A of liver function and an ECOG PS score of 0 before treatment. All the cases met the AASLD clinical diagnostic criteria for HCC, and six of them were pathologically proven to have HCC by biopsy. Seven of the patients had grade VP4 PVTT, two patients had grade VP3, and one patient had grade VP2 PVTT accompanied by inferior vena cava (IVC) invasion. One case also exhibited left hepatic vein invasion (18). Detailed demographic data are listed in **Table 1**.

Nine of the included patients were treated with four to six cycles of PD-1 inhibitor with TKI agents, and one patient received 10 cycles of the same treatment. All adverse events (AEs) associated with the combination therapy were evaluated and recorded in accordance with the National Cancer Institute (NCI) general term for adverse events (Common Terminology Criteria for Adverse Events, CTCAE) version 5.0. None of the patients presented with AEs or serious adverse events (SAEs) above CTCAE level 3.

### Radiologic Assessment

**Figure 1:**
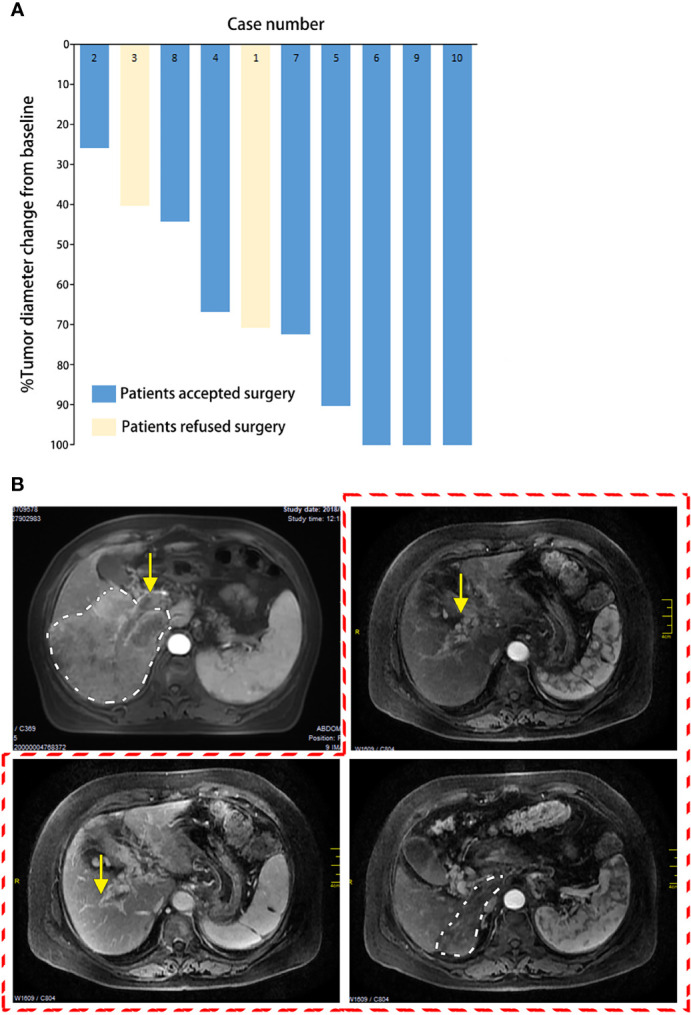
Tumor shrinkage after the conversion therapy. **(A)** Waterfall plot of maximum tumor shrinkage based on the modified response evaluation criteria in solid tumors (mRECIST) per independent imaging review. **(B)** Radiologic change of case no. 1. Before treatment (*first image outside the red dotted line*): tumor located in the right liver (outlined in *white dotted line*) and portal vein tumor thrombus (PVTT) in the right portal vein (*yellow arrow*). After treatment (outlined in *red dotted line*): tumor shrinkage (outlined in *white dotted line*), future liver remnant (FLR) enlargement, cavernous transformation of portal vein, and recanalization of the intrahepatic portal vein (*yellow arrow*).

At the first evaluation after the initiation of the combination therapy (after three cycles of immunotherapy), all patients had a significant reduction in tumor target lesion(s), accompanied by liquefactive necrosis or coagulative necrosis of the tumor(s). According to the mRECIST standard evaluation, PR was recorded in seven of 10 patients and in three patients with a complete response (CR) before salvage surgery (shown in **Table 1**). The complete necrosis rate of PVTT often precedes that of the primary tumor. In case no. 1, cavernous transformation of the portal vein (CTPV) and recanalization of the intrahepatic portal vein were observed in addition to dramatic enlargement of the remnant liver (shown in **Figure 1**).

**Figure 2:**
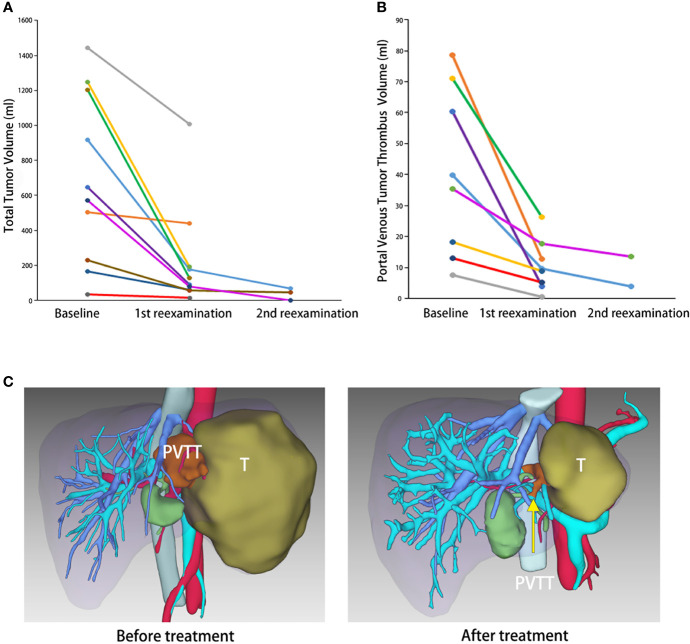
The total tumor volume (TTV) and portal vein tumor thrombus volume (PVTTv) change demonstrated by 3D reconstruction. **(A)** Change in TTV over time by 3D reconstruction. **(B)** Change in PVTTv over time by 3D reconstruction. **(C)** Three-dimensional reconstruction of case no. 4 before and after the combination therapy. *T*, tumor. *Yellow arrow*: PVTT.

Three-dimensional reconstruction was utilized to quantitatively analyze the TTV, PVTT, and FLR before and after treatment. TTV decreased by an average of 357.29 and 4.15 ml/day, with a peak of 21.97 ml/day (accounting for 1.48%/day of the standard liver volume). The PVTT shrank by an average of 27.34 and 0.29 ml/day, with a peak of 56.27 ml/day. Radiologic features such as coagulative necrosis, poor blood supply, and size reduction can also be observed in PVTT after treatment. Although the tumor-free liver volume increased enormously, the FLR was not obviously elevated in the quantitative evaluation due to the software settings, as the volume of the remaining liver after hemihepatectomy where the tumor was located (shown in **Table 2** and **Figure 2**).

**Table 2:** Change in tumor characteristics before *vs*. after the combined therapy.

	Patient/case no.									
	1	2	3	4	5	6	7	8	9	10
**ΔTTV (ml)**	−739.7	−60.9	−433.9	−556.5	−1,054.5	−105.5	−170	−18.8	−1,072.7	−494.37
**Time (days)**	145	84	43	97	48	107	86	93	75	61
**ΔTTV/day**	−5.1	−0.73	−10.09	−5.74	−21.97	−0.99	−1.98	−0.2	−14.3	−8.1
ΔTTV/SLV*day	−0.48%	−0.06%	−0.77%	−0.43%	−1.48%	−0.07%	−0.17%	−0.02%	−1.21%	−0.58%
**ΔFRLV (ml)**	35.8	32.1	−2.1	−41.8	98.2	2.02	−70	−145	35.1	0
**ΔFRLV/day**	0.25	0.38	−0.05	−0.43	2.05	0.02	−0.81	−1.56	0.47	0
ΔFRLV/SLV*day	0.02%	0.03%	0.00%	−0.03%	0.14%	0.00%	−0.07%	−0.14%	0.04%	0.00%
**ΔPVTTv (ml)**	−30.07	−65.9	−6.9	−56.27	−9.35	–	–	−7.75	−44.89	−17.61
**ΔPVTTv/day**	−0.21	−0.78	−0.16	−0.58	−0.19	–	–	−0.08	−0.6	−0.29

Estimated standard liver volume (SLV) calculated using the Urata formula: SLV = 706.2 × BSA + 2.4. Body surface area (BSA) calculated using the DuBois formula: BSA (m^2^) = BW (kg) 0.425 × BH (cm) 0.725 × 0.007184. Preoperative height (BH, measured to the nearest 1 cm), body weight (BW, measured to the nearest 1 kg).

FRLV, functional residue liver volume; PVTTv, portal vein tumor thrombus volume; SLV, standard liver volume; TTV, total tumor volume.

### Perioperative Information

The preoperative surgical evaluation took place after informed consent was obtained at the time the patient met the criteria for successful conversion. All patients in this study were successfully converted to surgery candidates. Two patients decided to continue to rely on the combination therapy protocol and eight patients underwent salvage surgery in our center. Surgical options included right hepatectomy, left hepatectomy, and anatomic segmental hepatectomy, depending on the patient’s tumor localization, degree of cirrhosis, and liver reserve. The perioperative information is listed in **Table 3**. **Figure 2** shows a typical case who had the combination therapy and underwent subsequent surgery (case no. 6).

**Table 3:** Operative and pathological information.

	Patient/case no.									
	1	2	3	4	5	6	7	8	9	10
Preoperative Child–Pugh grade	–	A	–	A	A	A	A	A	A	A
Preoperative ECOG PS score	–	0	–	0	0	0	0	0	0	0
Preoperative AFP (ng/ml)		1,673.5		1.7	323.1	5.1	2.5	2.2	2,198.3	127.6
Operation type	–	Right hepatectomy	–	Left hepatectomy	Right hepatectomy	Laparoscopic liver resection of S4, 5, 8	Liver resection of S5, 8	Right hepatectomy	Right hepatectomy	Left hepatectomy
Operation time	–	4 h, 25 min	–	4 h, 20 min	5 h, 50 min	4 h, 35 min	4 h, 23 min	3 h, 50 min	4 h, 20 min	5 h, 56 min
Blood loss (ml)	–	400	–	50	3,000	100	500	2,400	1,700	2,000
Blood transfusion	–	Autologous blood transfusion 2 U	–	–	RBC 2 U; plasma, 4.5 U	–	–	RBC, 2 U; plasma, 2.4 U	RBC, 4 U, plasma, 3.8 U	RBC, 4 U; plasma, 1.1 U
Mechanical ventilation time (days)	–	1	–	1	1	1	1	1	1	1
Abdominal drainage time (days)	–	6	–	5	9	5	8	8	7	7
Fasting time (days)	–	2	–	3	4	3	4	3	3	4
mRECIST before surgery	PR	PR	PR	PR	PR	CR	PR	PR	CR	CR
Viable tumor cell rate	–	90%	–	3%	1.5%	2.5%	<5% and 50% (for different lesions)	100%	15%	0% (pCR)

CR, complete response; mRECIST, modified respond evaluation criteria solid tumors; pCR, pathological complete response; PR, partial response; RBC, red blood cell.

In this case series, the interval between lenvatinib withdrawal and surgery was 3–7 days. The mean intraoperative blood loss was 1,650 ml (50–3000 ml). Endotracheal intubation was removed in all patients within 24 h. There was no postoperative mortality. There was no postoperative bleeding or bile leakage and no other postoperative complications beyond Clavien–Dindo level III (requiring surgery, endoscopy, or interventional intervention). The liver function indexes, such as transaminase and bilirubin, returned to normal within 7–10 days after surgery (within two times the upper limit of normal, ULN). All patients were discharged from the hospital within 10 days after surgery after successfully removing the abdominal drainage tube. Some of the patients suffered from postoperative elevated transaminase, hypoproteinemia, serosal effusion, and other manifestations of liver dysfunction, which could be significantly improved by conservative measures, such as both early enteral and parenteral nutrition.

### Pathology and Postoperative Maintenance Treatment

**Figure 3:**
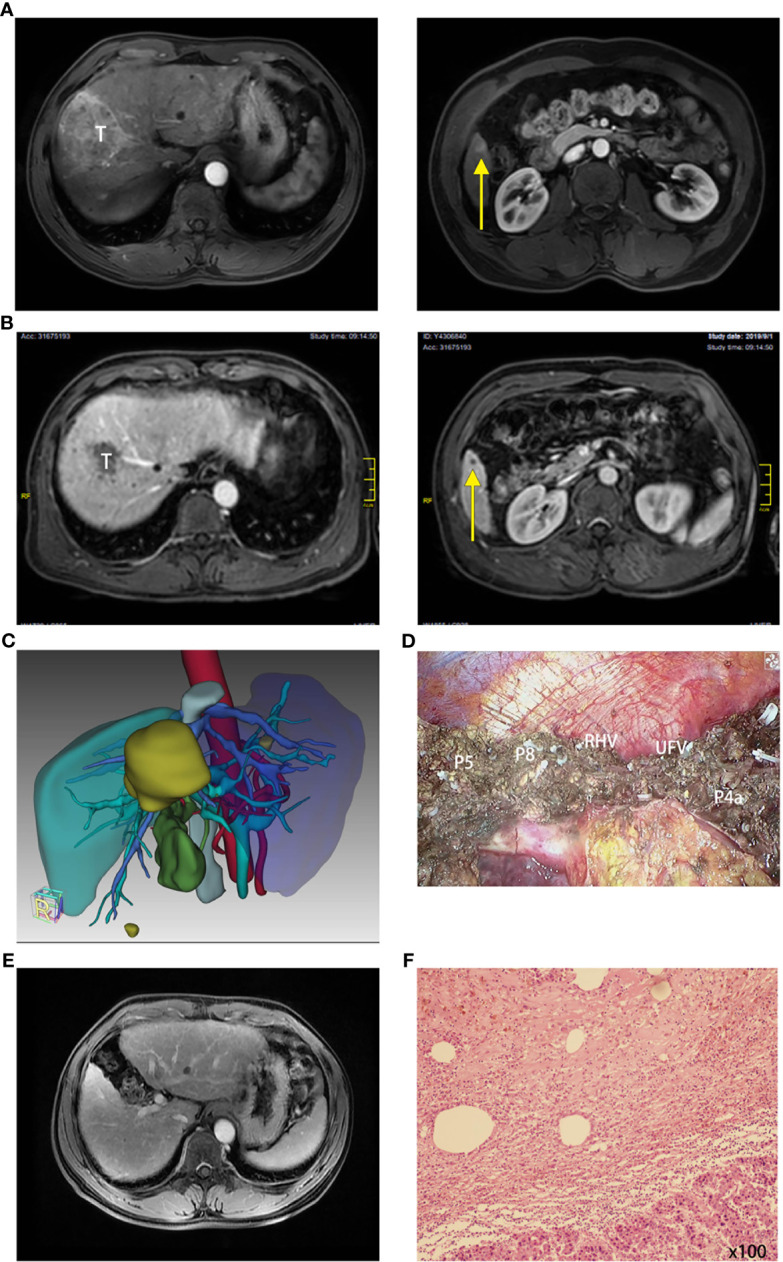
A typical case had the combination therapy and underwent subsequent surgery (case no. 6). **(A)** Radiology of case no. 6 before treatment: *T* for tumor 1 and *yellow arrow* pointing tumor 2. **(B)** Radiology of case no. 6 after treatment: *T* for tumor 1 and *yellow arrow* pointing tumor 2. **(C)** Three-dimensional reconstruction and surgical plan of case no. 6 after the combination therapy. **(D)** Surgical photo of the margin (*P* for pedicle, *RHV* for right hepatic vein, and *UFV* for umbilical fissure vein). **(E)** Reexamination of the liver 6 months after the surgery: No tumor or relapse. **(F)** Pathology of tumor: moderately differentiated hepatocellular carcinoma (HCC) is focally distributed with extensive necrosis and several inflammatory cells (×100); viable tumor cell rate, 2.5%.

Surgical specimens (such as tumor lesions and tumor thrombi) were analyzed by pathologists. Negative margins were achieved in all cases. The rate of residual viable tumor cells was reported in detail for every case. Residual viable tumor cells in the tumor thrombus were found in two of six cases with PVTT grade VP3/VP4. The rate of viable tumor cells of the PR cases (based on mRECIST) varied from 1.5% to 100%, as shown in **Table 3** and **Figure 3**. Only patient no. 10 had pathology-proven pathological complete response (pCR) among the three patients who achieved CR (using mRECIST), and sporadic viable tumor foci were found in specimens of the other two cases (see **Table 3** and **Figure 3**).

The combined therapy of an anti-PD-1 inhibitor and TKIs was continued for at least six cycles after surgery, except for the pCR case who was suggested a PD-1 inhibitor administration once every 3 weeks for at least six cycles. Every patient underwent reexamination 1–3 months after surgery.

### Postoperative Follow-Up and Long-Term Survival

**Figure 4:**
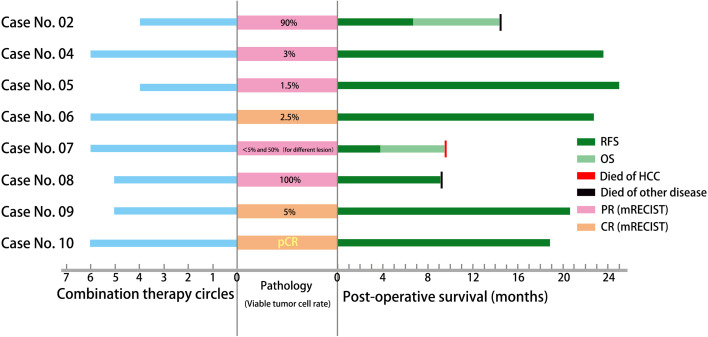
General view of combination therapy, surgery, pathology, and post-surgery survival.

All the patients who underwent salvage surgery were well followed up, and the median follow-up time was 19.7 months (9.1–24.9 months). In all cases at the first visit. no tumor was found radiologically, and the AFP values were reduced to normal. But relapse occurred in case no. 2 (6.8 months after operation) and case no. 7 (3.8 months after operation), and the latter died of HCC 9.5 months postoperatively. Case no. 2 died of acute cholangitis and septic shock 14.4 months after surgery. Case no. 8 presented with advanced lung cancer (pathology-confirmed) and died 9.1 months postoperatively. The 12-month RFS rate of all cases who underwent salvage surgery was 75% (**Figure 4**).

## Discussion

### The Possibility of Immunotherapy Combined with Targeted Therapy as a Conversion Therapy

Based on the large population of advanced HCC in East Asia, some hepatobiliary institutions in Asia have performed surgical resection for a broader spectrum of advanced-stage HCC patients, but the long-term survival is still unsatisfactory (19, 20). We noted that both neoadjuvant therapy and conversion therapy have been successfully performed for colorectal cancer liver metastasis (CRLM) and locally advanced pancreatic cancer (21–25). Therefore, we initiated the conversion therapy scheme for unresectable HCC, stated above.

**Figure 5:**
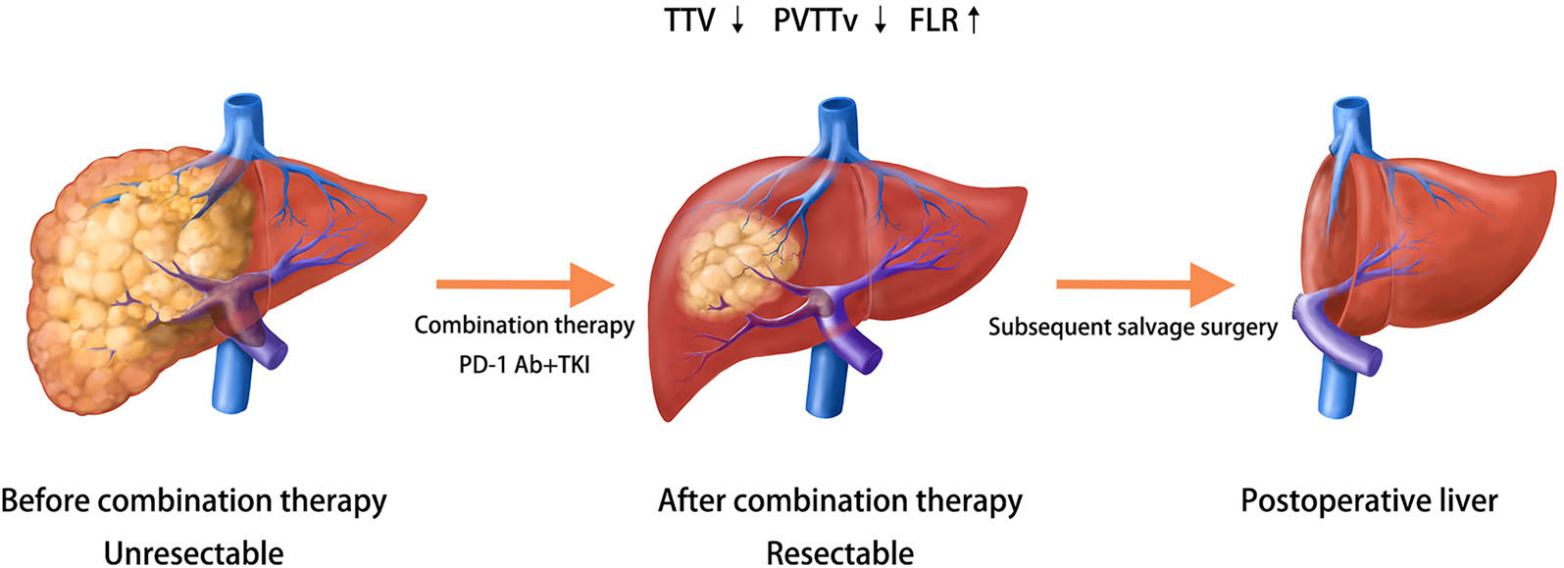
Possibility of immunotherapy combined with targeted therapy as a conversion therapy.

After the combination therapy of immunotherapy with TKI agents, the cases with objective response showed obvious tumor shrinkage with or without PVTT shrinkage and even FLR enlargement in reported clinical trials and case series in our center, which is noninvasive and safe with only a few AEs. When we evaluated the antitumor treatment, some of the patients had met the criteria to indicate surgery. Therefore, combination therapy may be promising as an effective and efficient conversion therapy protocol for advanced HCC, as shown in **Figure 5**.

### Methods for Evaluating the Feasibility of Surgery

Both morphological and functional examinations need to be undertaken in order to estimate the therapeutic effect before salvage surgery. Both RECST1.1 and mRECIST were deemed feasible for the therapeutic evaluation of advanced HCC. The RECIST 1.1 criteria were intuitive and favorable for surgical decision-making, but only considered tumor shrinkage in a single diameter. mRECIST may be consistent with pathology and oncology better (17).

Our group introduced the method of quantitative oncology based on imaging (CT/MRI) in the evaluation process. The reductions in TTV and PVTT volume and the increase in FLR were evaluated and shown more individually and clearly. The integrity of the inflow and outflow of the reserved liver can be considered as an important criterion for successful conversion because PVTT necrosis also prevents intrahepatic metastasis through the portal vein. Combined with other liver reserve function tests, such as the indocyanine green (ICG) clearance test (26, 27), and general status assessment, such as the ECOG PS score, we could determine the feasibility and safety of large-volume hepatectomy after conversion therapy.

### Timing of Surgery

How to determine the time of surgery is still under discussion: early operation may be risky and fail to take full advantage of the effects of the combination therapy, whereas secondary drug resistance and tumor progression may occur before late operation. It has been reported that most cases reach peak efficacy after three to six cycles of the treatment protocol (28, 29). As a result, we suggest that salvage surgeries can be performed after four to six cycles of combination therapy for PR or CR cases if the patient meets the surgery criteria listed in *Method*.

In the past, in the clinical practice of “neoadjuvant therapy” for CRLM, it was customary to perform surgery after the anti-angiogenic drugs were discontinued for a period of time to prevent intraoperative bleeding and poor healing (30, 31). In this case series, the interval between lenvatinib withdrawal and surgery was 3–7 days according to the half-life period data. There were no perioperative bleeding complications in all cases despite an increased number of transfusions. Poor wound healing and secondary suture were found in one case. However, this patient had significant hypoproteinemia secondary to decompensated liver function, which occurs frequently in cirrhotic subjects after surgery, and was considered the main reason for the poor wound healing.

### Pathology: Accurate Response Evaluation and Predictor of Long-Term Survival?

In this case series, there seemed to be some discrepancy between the radiologic evaluation and pathology. We had three radiologic CR cases, but only one pCR, which was defined as: if no viable tumor cell found by pathology and patients with PR also had different percentages of viable tumor cells.

This could be attributed to an inadequate duration of the combined therapy, but further reflected the low response rate of the remaining tumors to therapy. There are two aspects that may result to this. Firstly, each HCC is composed of a unique combination of somatic alterations, including genetic, epigenetic, transcriptomic, and metabolic events, that form its unique molecular fingerprint, which is the underlying cause of the heterogeneity in HCC (32, 33). Moreover, tolerance to immunotherapy may be induced by T-cell exhaustion due to the absence of an effector T-cell response to neoantigens in some metastases, as a result of immunotherapy (21, 34). Regardless of the cause, inaccurate imaging assessment can lead to misjudgment of the next treatment choice. Therefore, we suggest surgery for all patients who meet the surgery criteria because: 1) pathology is the gold standard for response evaluation and 2) surgery is probably an effective measure to eliminate viable tumors caused by heterogeneity or secondary resistance to the combined therapy.

Furthermore, it seems that patients with a high viable tumor cell rate had worse prognosis, which means that pathology could be a predictor of long-term survival and offer information for the next step of treatment of patients. The small number of cases in this study was insufficient to draw conclusions with a higher level of evidence. Further prospective studies with a larger sample size are needed to confirm this.

### Maintenance of Postoperative Antitumor Therapy

After a series of multidisciplinary treatment discussions focusing on the maintenance of postoperative antitumor therapy and sufficient informed consent, all patients continued using the PD-1 inhibitor for at least 6 months for the following reasons: as the body’s antitumor immunity is activated, participants in antitumor immunity, such as CD8^+^ T cells, tumor-associated macrophages (TAMs), and natural killer (NK) cells, exist not only in the tumor immune microenvironment but also in peripheral circulating blood. This is the intrinsic mechanism of the antitumor effect of immunotherapy. Among them, memory T-cell recruitment within the body plays a critical role in the long-term maintenance of the antitumor cytotoxic effects during postoperative immunological surveillance (35, 36). Therefore, continuous PD-1 inhibitor administration after surgery may elicit immunity against potential residual viable tumor cells, which was proven to be the origin of recurrence, as reported elsewhere in the literature (37). Patients for whom the pathology reported viable tumor cells should continue the lenvatinib therapy starting 1 month after surgery and last no less than 6 months.

## Conclusion

The preliminary results of this case series suggest that immunotherapy based on PD-1 inhibitor combined with TKIs could be a reasonable and promising conversion therapy for advanced HCC with major vascular invasion. Salvage surgery was effective and safe after conversion therapy with PD-1 inhibitors plus TKIs for advanced HCC, and a combination therapy protocol including surgical treatment may increase the long-term oncological benefit.

However, this needs more prospective clinical trials in order to provide higher level evidence and discuss the preoperative radiologic assessment, pathology evaluation, and postoperative systematic treatment.

## Data Availability Statement

The original contributions presented in the study are included in the article/supplementary material. Further inquiries can be directed to the corresponding authors.

## Ethics Statement

The studies involving human participants were reviewed and approved by the Medical Ethics Committee of PLA General Hospital. The patients/participants provided written informed consent to participate in this study. Written informed consent was obtained from the individual(s) for the publication of any potentially identifiable images or data included in this article.

## Author Contributions

SL was responsible for concept and design. SL and HW provided administrative support. SL, BH, HW, WZ, and JH performed surgery. WZ, JH, JC, ZZ, JS, MC, XW, YC, YX, and LT collected and assembled the data. WZ, JH, BH, ZW, GM, HY, JY, and ZZ analyzed and interpreted the data. WZ, JH, and SL prepared the manuscript. SL, WZ, and JH critically revised the manuscript. All authors critically reviewed or revised the manuscript for intellectual content and approved the final version to be submitted.

## Funding

This work was supported by the National Natural Science Foundation of China (no. 81670590) and the National Key Research and Development Program of China (2017YFA0103003).

## Conflict of Interest

The authors declare that the research was conducted in the absence of any commercial or financial relationships that could be construed as a potential conflict of interest.

The reviewer HZ declared a shared affiliation with one of the authors HW to the handling editor at the time of the review.

## Publisher’s Note

All claims expressed in this article are solely those of the authors and do not necessarily represent those of their affiliated organizations, or those of the publisher, the editors and the reviewers. Any product that may be evaluated in this article, or claim that may be made by its manufacturer, is not guaranteed or endorsed by the publisher.
